# Bioengineered Kidney Tubules Efficiently Clear Uremic Toxins in Experimental Dialysis Conditions

**DOI:** 10.3390/ijms241512435

**Published:** 2023-08-04

**Authors:** João Faria, Sabbir Ahmed, Dimitrios Stamatialis, Marianne C. Verhaar, Rosalinde Masereeuw, Karin G. F. Gerritsen, Silvia M. Mihăilă

**Affiliations:** 1 Division of Pharmacology, Utrecht Institute for Pharmaceutical Sciences, Utrecht University, 3584 CG Utrecht, The Netherlands; j.p.ferreirafaria@uu.nl (J.F.); s.ahmed@uu.nl (S.A.); r.masereeuw@uu.nl (R.M.); 2 Advanced Organ Bioengineering and Therapeutics, Faculty of Science and Technology, Technical Medical Centre, University of Twente, 7522 NB Enschede, The Netherlands; d.stamatialis@utwente.nl; 3 Department of Nephrology and Hypertension, University Medical Center, 3508 GA Utrecht, The Netherlands; m.c.verhaar@umcutrecht.nl (M.C.V.); k.g.f.gerritsen@umcutrecht.nl (K.G.F.G.)

**Keywords:** bioartificial kidney, dialysis fluid, uremic plasma, uremic toxins, proximal tubule epithelial cells

## Abstract

Patients with end-stage kidney disease (ESKD) suffer from high levels of protein-bound uremic toxins (PBUTs) that contribute to various comorbidities. Conventional dialysis methods are ineffective in removing these PBUTs. A potential solution could be offered by a bioartificial kidney (BAK) composed of porous membranes covered by proximal tubule epithelial cells (PTECs) that actively secrete PBUTs. However, BAK development is currently being hampered by a lack of knowledge regarding the cytocompatibility of the dialysis fluid (DF) that comes in contact with the PTECs. Here, we conducted a comprehensive functional assessment of the DF on human conditionally immortalized PTECs (ciPTECs) cultured as monolayers in well plates, on Transwell^®^ inserts, or on hollow fiber membranes (HFMs) that form functional units of a BAK. We evaluated cell viability markers, monolayer integrity, and PBUT clearance. Our results show that exposure to DF did not affect ciPTECs’ viability, membrane integrity, or function. Seven anionic PBUTs were efficiently cleared from the perfusion fluid containing a PBUTs cocktail or uremic plasma, an effect which was enhanced in the presence of albumin. Overall, our findings support that the DF is cytocompatible and does not compromise ciPTECs function, paving the way for further advancements in BAK development and its potential clinical application.

## 1. Introduction

Chronic kidney disease (CKD) is a progressive condition characterized by the deterioration of kidney function, ultimately leading to end-stage kidney disease (ESKD). As kidney function declines, endogenous metabolites that are normally cleared by the kidney accumulate in the plasma of CKD patients. These metabolites are referred to as uremic retention solutes, and their accumulation can lead to cellular dysfunction, inflammation, and oxidative stress [1], all of which are associated with CKD-related comorbidities such as cardiovascular disease, secondary immunodeficiency, and neurologic manifestations, and to the progression of CKD itself [2]. Uremic retention solutes are classified based on their physicochemical properties and dialytic removal patterns [2]. Among these uremic retention solutes, protein-bound uremic toxins (PBUTs) are of particular concern, as they have a strong binding affinity towards plasma proteins, in particular albumin, and are not effectively removed by conventional dialysis methods [3].

To address the challenge of enhancing PBUTs clearance, we drew inspiration from the natural process of PBUTs elimination within the kidneys. Proximal tubule epithelial cells (PTECs) in the kidney express key membrane transporters, the organic anion transporters (OATs), which are responsible for the secretion of anionic PBUTs from the blood into urine [4]. OAT1 (*SLC22A6*) and its homolog OAT3 (*SLC22A*8) are the most important transporters involved in the uptake and excretion of a variety of endogenous metabolites, including PBUTs, but also drugs administered to patients, with OAT1 having a higher capacity for handling the smaller compounds [4,5,6]. Incorporating an active secretory compartment composed of PTECs into the development of a bioartificial kidney (BAK) device would facilitate the efficient removal of PBUTs. Previously, we designed a BAK using porous hollow fiber membranes (HFMs) covered with a monolayer of conditionally immortalized proximal tubule cells (ciPTECs) on the outer surface [7]. In this configuration, the cells demonstrated the expression and function of the basolateral OAT1 and an effective removal of the PBUTs’ kynurenic acid (KA) and indoxyl sulfate (IS) [7].

To ensure the clinical suitability of the BAK, it is of paramount importance to determine whether these bioengineered tubules can effectively secrete PBUTs under conditions mimicking their intended use, viz. as an extracorporeal device connected in series with a conventional hemodialysis filter. In this setup, blood is pumped through the dialyzer (hemofilter or BAK), while dialysis fluid (DF) is perfused on the other side of the membranes, as described by Ramada et al. [8]. DF is an essential component in standard dialysis, as it facilitates the purification process and enables the removal of harmful substances from the body [9]. Given the direct exposure of cells to DF, it is essential to assess its effects on cellular homeostasis. Therefore, the aim of this study was to evaluate the function of bioengineered kidney tubules under experimental dialysis conditions resembling those encountered in the intended application of BAK. Specifically, we examined cell viability markers following DF exposure, assessed the effects of the DF in combination with healthy and uremic plasma (HP and UP, respectively) on the “blood side” on the monolayer integrity using a Transwell^®^ system, and investigated the potential of bioengineered kidney tubules for PBUTs clearance during exposure to DF. We perfused these tubules with UP at the basal side and DF at the apical side to simulate experimental dialysis conditions in patients with ESKD.

## 2. Results

### 2.1. Exposure of ciPTECs to DF in 2D Leads to Minor Alteration in Cell Viability Markers

**Figure 1:**
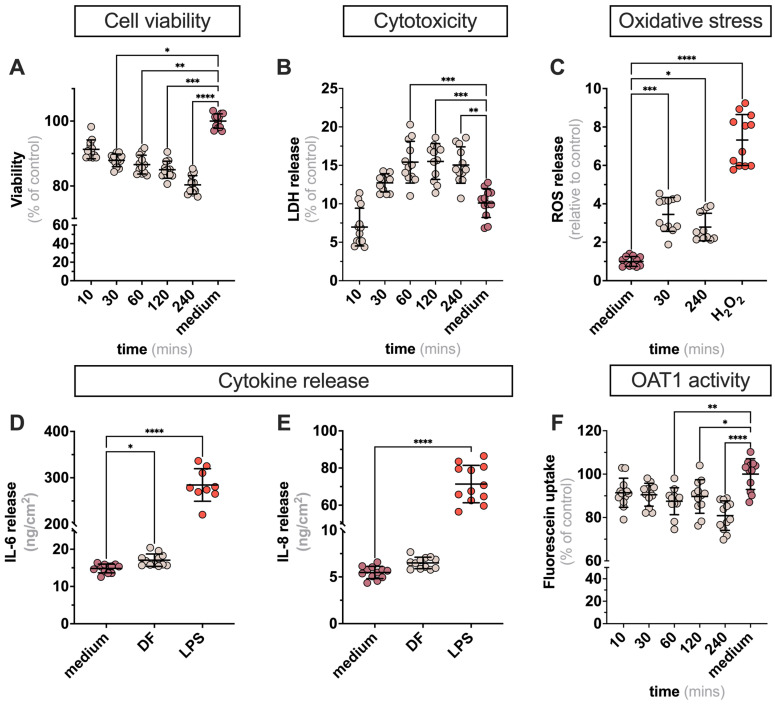
**Characterization of ciPTECs upon exposure to DF.** To assess the effect of DF on cell behavior, measurements of cell viability (**A**), cytotoxicity (**B**), oxidative stress (**C**), cytokine release (**D**,**E**), and OAT1 activity (**F**) were performed on ciPTECs-OAT1. H_2_O_2_ (500 µM) and LPS (1 μg/mL) were used as positive controls to oxidative stress and cytokine release, respectively. Data are shown as mean ± SD of four replicates from four independent experiments. * *p <* 0.01, ** *p <* 0.01, *** *p <* 0.005, and **** *p <* 0.0001 using a one-way ANOVA analysis followed by Tukey’s multiple comparisons test.

To investigate whether the DF affects cell homeostasis, ciPTECs overexpressing OAT1 (ciPTECs-OAT1) were exposed to DF (see Supplementary Table S1 for composition) or medium (control) for up to 240 min, the standard time of a dialysis session (Figure 1A). After 30 min of exposure to DF, the cell viability decreased slightly. This descending pattern was maintained over time reaching 80 ± 4% of control (Figure 1B), which is the threshold of any intervention to be considered as noncytotoxic [10]. Furthermore, the release of LDH, a marker of plasma membrane damage, increased during the first 60 min but remained stable for the remainder of the exposure time (Figure 1C). This was accompanied by an increase in intracellular ROS production, an indicator of oxidative stress (Figure 1D). The release of IL-6 and IL-8 was not affected (Figure 1E,F), suggesting that exposure to DF does not elicit an inflammatory response. We next investigated the activity of OAT1, the key transporter involved in the secretion of PBUTs, through the uptake of fluorescein as substrate [11]. We found that the OAT1-mediated uptake capacity slightly decreased over time, in line with the decrease in cell viability. Overall, these findings suggest that exposure of ciPTECs-OAT1 to DF for 4 h does not warrant concerns in terms of viability and functionality.

### 2.2. CiPTECs-OAT1 Cultured on Flat Membranes Show Minor Changes in Barrier Integrity upon Exposure to DF (Apically) and Plasma (Basolaterally)

**Figure 2:**
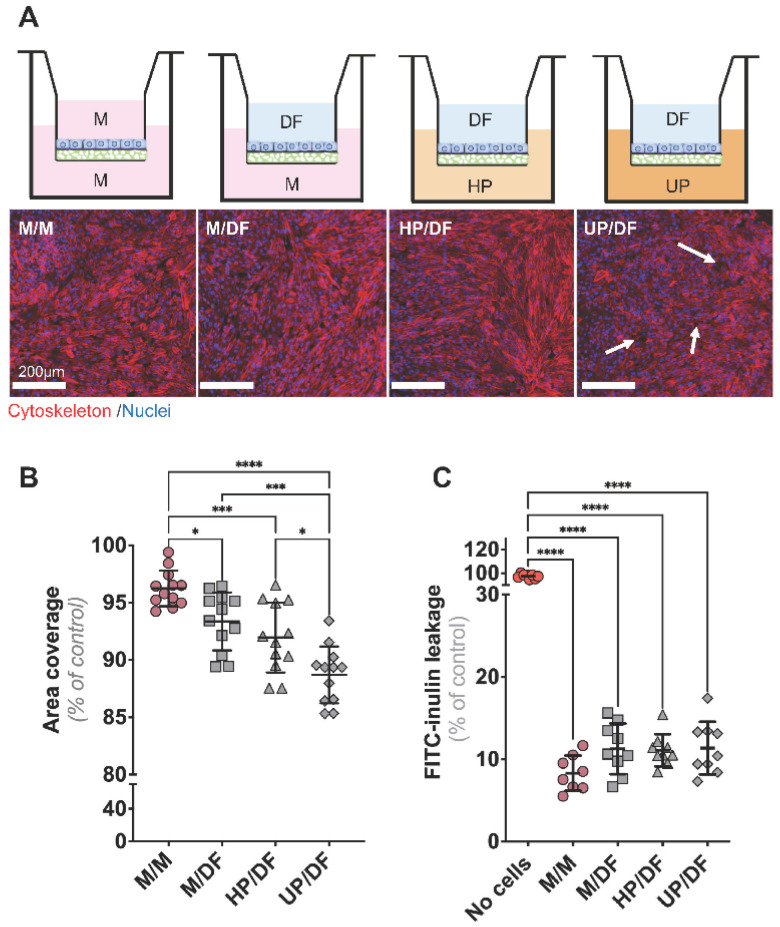
**Evaluation of the monolayer integrity in flat membranes exposed to DF and plasma.** (**A**) F-actin filaments (in red) and nuclei (in blue) were stained with phalloidin and DAPI, respectively, to assess damage in the cell monolayer for different experimental settings, as depicted by the schematics. White arrows indicate monolayer derangements. (**B**) Measurement of cell surface coverage in the different experimental settings. (**C**) Monolayer permeability was evaluated by the leakage of FITC-inulin from the basal to apical compartment. Data are shown as mean ± SD of four replicates from three independent experiments. * *p <* 0.01, *** *p <* 0.005, and **** *p <* 0.0001 using a one-way ANOVA analysis followed by Tukey’s multiple comparisons test. Abbreviations: M, medium; DF, dialysis fluid; HP, healthy plasma; UP, uremic plasma.

Next, we investigated the effect on the monolayer integrity of a simultaneous exposure for 4 h to plasma at the basal side and DF at the apical side of the cell-covered membranes. Staining for the cytoskeleton (phalloidin) allowed us to visualize the coverage of the cell monolayer on the porous surface (Figure 2A). Cells cultured with medium in both apical and basal compartments displayed a full coverage of the membrane (Figure 2A,B). With the addition of DF to the apical side, the coverage percentage slightly decreased (Figure 2B). This trend became more evident when HP or UP, respectively, were added to the basal side, with small cell-free areas emerging in the monolayer (Figure 2A,B). Furthermore, the observed gaps in the monolayer did not result in fluid flowing through the gaps. We then analyzed the barrier properties of cell-seeded membranes under the same conditions by applying FITC-inulin, a commonly used tracer to assess the monolayer integrity, at the basal side (Figure 2C). Despite the loss in cell coverage under apical exposure to DF (Figure 2B), the barrier properties were not affected, as the leakage of FITC-inulin at the apical side was not enhanced in the aforementioned conditions (Figure 2C).

### 2.3. CiPTECs-Seeded HFMs Effectively Secreted PBUTs when Exposed to Plasma

**Figure 3:**
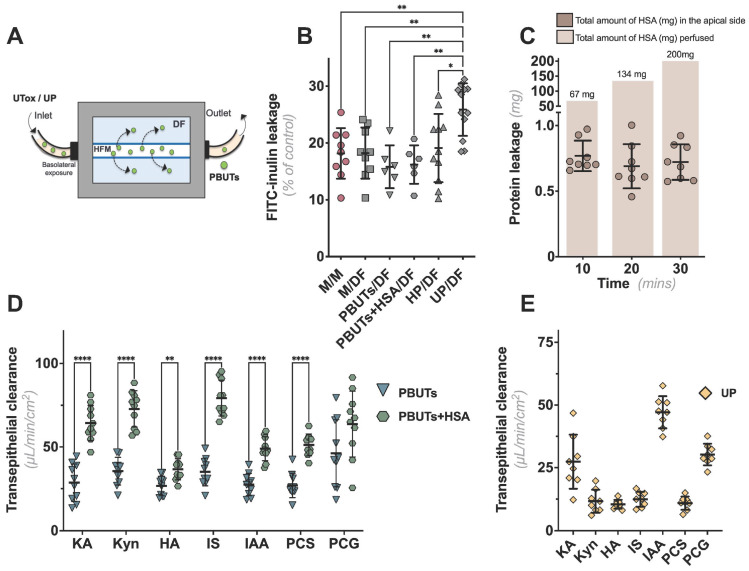
**Evaluation of the monolayer integrity in HFM upon exposure to DF and UP**. (**A**) Schematic representation of the BAK prototype. (**B**) Upon cell maturation, cells were perfused with either medium, PBUTs, PBUTs with HSA (PBUTs + HSA), HP, or UP for 30 min, followed by a 10 min perfusion with FITC-Inulin to evaluate monolayer integrity. (**C**) Quantification of total HSA leakage at the apical side. In brown blocks is the total amount of HSA (mg) perfused (6 mL/h) through the fibers at the corresponding time points. (**D**) Transepithelial clearance of PBUTs during the perfusion of the fibers with either PBUTs alone, with HSA 1 mM, or (**E**) UP. Data are shown as mean ± SD of four replicates from three independent experiments. * *p <* 0.01, ** *p <* 0.01, and **** *p <* 0.0001 using (**A**) a one-way ANOVA analysis followed by Tukey’s multiple comparison tests or (**D**) multiple Mann–Whitney tests. Abbreviations: M, medium; DF, dialysis fluid; HP, healthy plasma; UP, uremic plasma; KA, kynurenic acid; Kyn, kynurenine; HA, hippuric acid; IS, indoxyl sulfate; IAA, indole-3 acetic acid; PCS, p-cresyl sulfate; PCG, p-cresyl glucuronide.

We previously developed bioengineered kidney tubules using an HFM system as functional units for a BAK in which the cells are seeded on the outer surface of a double-coated fiber [12]. To replicate the intended use, we perfused the lumen of the ciPTECs-seeded HFMs with perfusates containing PBUTs for 30 min by using a custom-made flow system (Figure 3A). The addition of DF to the apical compartment did not affect the barrier performance of the cell monolayers, as determined by the FITC-inulin leakage, regardless of the perfusates composition (Figure 3B). To confirm the barrier function towards albumin, we collected samples from the apical compartment after basolateral perfusion with a buffer containing human serum albumin (HSA; 1 mM) at specific time points. During the first 10 min of perfusion, only 1% of albumin was lost, which was maintained during the rest of the experiment (Figure 3C). These findings not only support active albumin reabsorption but also show that our monolayer prevents substantial protein loss under physiological conditions.

We next evaluated the efficacy of the bioengineered kidney tubules to actively remove PBUTs (Figure 3D). Transepithelial transport, from basal to apical side, was determined for seven PBUTs and quantified using LC-MS/MS to calculate their clearance rates. Notably, we built upon our previous knowledge on PBUT clearance, in a probenecid-sensitive manner [12,13,14]. However, in the current study, we did not include this control to emphasize the pivotal role of our bioengineered kidney tubules in effectively removing PBUTs. All PBUTs tested were detected at the apical side in either PBUT or PBUT+HSA basolateral perfusions. While the first perfusate displayed comparable clearance rates for all UTs, the presence of HSA led to a significant increase in clearance rates, especially for kynurenic acid (KA), kynurenine (Kyn), and indoxyl sulfate (IS) (Figure 3D). In addition, as a proof of concept, we investigated the transepithelial clearance of the same PBUTs in an experimental model that best reflected the use of the BAK system by perfusing with patient-derived UP with known PBUTs concentrations (Supplementary Table S2) and apical exposure to DF (Figure 3D). Except for indole-3 acetic acid (IAA), perfusing kidney tubules with UP resulted in lower transepithelial clearance rates than perfusing with PBUTs, despite the presence of HSA (Figure 3E). Table 1 summarizes the comparisons of the different perfusion settings and their respective clearance rates.

## 3. Discussion

The development of a BAK as a viable addition to traditional dialysis therapies for ESKD patients is an ongoing research endeavor. A key component of such device is a “living membrane” that consists of a PTEC monolayer on a porous membrane that allows the transport of molecules from one side to the other. Previous BAK research has focused on determining its feasibility, safety, effectiveness, and design optimization [15,16,17]. While these characteristics are important for clinical translation, there is still a lack of information regarding the cytocompatibility of the DF, a solution that will be in direct contact with the BAK-containing cells. To address this, we conducted a study in which PTECs, highly appealing for BAK-like applications, were cultured on porous membranes and exposed to DF at the apical side and plasma at the basal side, a representation of the setup envisioned for BAK. Our findings demonstrate acceptable viability and active transepithelial PBUT clearance when PTEC monolayers were exposed to the DF.

In our study, we initially assessed a panel of cell viability parameters by exposing ciPTECs to DF for a period of up to 4 h, the standard duration of a dialysis session. We observed that exposure to DF did not induce cytotoxicity or inflammation in flat cultures. However, we noted a minor decrease in cell viability and OAT1 activity, suggesting minimal damage or environmental changes that could compromise its function. We did observe an initial increase in ROS production likely due to an acute response to the DF, which subsided over time. This could be caused by a lack of nutrients or adverse effects to DF components such as acetate, whose concentration in DF is higher than what cells would encounter in cell culture medium [18,19,20]. Instead of DF, the use of culture medium during HD could be considered to provide a more stable environment for the cells, thus supporting its viability and function [21]. However, this coincides with increased treatment costs and is more challenging to control for batch-to-batch variation in some of the components of culture medium. The addition of specific components to the DF, such as amino acids and growth factors, could prove to be more beneficial for BAK applications. Still, considering the limited magnitude of the adverse effects within the allowable range, we conclude that the DF can be used in close contact with cells for the duration of a dialysis session without causing significant problems.

Another key parameter investigated in our study was the combined effect of the DF and plasma on the integrity of the PTEC monolayer, thereby mimicking the BAK application setting. Exposure to both DF and UP resulted in a minor disruption of the monolayer, most likely due to a modulation of the tight junctions [22]. This may be due to both the nutrient-deprived nature of the DF and the complex composition of UP, which contains many other toxins and drugs prescribed to ESKD patients. The combination of these factors creates a more toxic environment for the cells, resulting in the observed changes in monolayer integrity. Additionally, the possible presence of shed cells due to the exposure to DF could have also played a role in the observed changes. Furthermore, PBUTs found in UP have been shown to negatively impact cell function and phenotype [23], stimulate inflammatory pathways [24], and affect tight junctions [22], thus potentially compromising the barrier function and BAK efficacy.

To create a model relevant to BAK setup, we employed the HFMs-based system. As already reported for the 2D model, the barrier function was maintained when the bioengineered tubules were concomitantly exposed to PBUTs (alone or with HSA) and DF. Additionally, we evaluated the efficacy of the HFM-based BAK model in removing PBUTs. Our data revealed that all tested PBUTs demonstrated a higher transepithelial clearance in the presence of HSA, pointing towards the potential role of albumin as a facilitator of the transporter activity, an observation noted earlier for transport in kidney [7,25] but also liver [26,27]. When exposed to UP, with a more complex composition in terms of uremic compounds’ load, including PBUTs’ repertoire, ciPTECs were still able to remove PBUTs; however, the clearance rate was lower compared to experimentally controlled uremic conditions. This discrepancy is in line with the complex composition of UP which can interfere with the overall cell capacity to clear these PBUTs. Additionally, the initial PBUTs’ concentrations in the experimental uremic conditions, chosen to reflect the average PBUTs’ concentrations reported in ESKD (https://database.uremic-toxins.org, accessed on 28 March 2023 and ref. [24]), are different than those we identified in the UP that we used in this study. Furthermore, since we measured the total concentration of PBUTs (both free and protein-bound), we cannot attribute the differences in clearance rates to partitioning between water and albumin. Instead, the variations in clearance rates are likely influenced by the individual characteristics of the PBUTs, such as initial concentration, protein binding properties [28] and OAT1 affinity [4]. In future studies, the partitioning between water and albumin should be addressed in order to provide a more accurate interpretation of the results. Nonetheless, while our model demonstrates the ability to remove PBUTs, the actual clearance capacity and effectiveness might vary per patient. Moreover, in future studies, several uremic plasma batches, with a known profile of PBUTs and medication load should be tested.

Dialysis patients are often prescribed drugs that have been shown to compete with the transporter function for PBUTs [13]. These patients do not share the same medical history and their residual kidney function, a determinant in toxin clearance, differs among them [29]. Furthermore, the PBUTs studied here are all OAT1 substrates while UP may also contain additional UTs [30,31] and other molecules that may impact the transporter activity and contribute to the patients’ clinical deterioration. Of note, Jansen et al. demonstrated that in short exposure, IS can specifically enhance the expression and function of OAT1 in ciPTECs through a complex, receptor-mediated process [32]. This was explained as a coordinated function through sensing increased levels and stimulating this PBUT’s clearance, adhering to the remote sensing and signaling theory [33]. Subsenquently, enhancing PBUTs’ clearance through a preconditioning approach, thereby activating signalling pathways such as the remote-sensing one, could allow for a reduction of the BAK size. Of note, a complete removal of PBUTs can also lead to adverse effects as these are also involved in metabolic processes [34].

Another important factor to consider is protein leakage, which compromises the patients’ prognosis. During dialysis, albumin loss is influenced by the type of dialysis membrane and different HD modalities. Notably, high-cut-off HD membranes, which are known for their high efficiency in solute removal in dialysis and similar to those used in this stuy, have been reported to result in an albumin loss with a range of 6–9 g/4–5 treatment [35]. Despite this, the acceptable limit of albumin loss for a potential BAK device has yet to be determined as this will differ among BAK’s design and patients with different medical records. In the present study, only 1% of albumin was lost during the 30 min of perfusion. This loss in protein may be due to the active reabsorption by the cells, as previously reported [7], or the albumin adsorption to the system [36,37]. Nevertheless, our results support that the cell monolayer in BAK prevents a substantial protein loss under physiological conditions.

While this study has several notable strengths, it also has some limitations. First, the lack of an apical, unidirectional flow during cell culture and dialysis may have influenced proximal tubule performance. Nonetheless, our current perfusion setup offers a simpler approach to culture cells on fibers and load them into the perfusion chambers. Compared to more complex microfluidic devices, our setup allows us to generate more robust, reliable and reproducible data. The addition of a fluid shear stress has been reported to support the proximal tubule’s phenotype by improving cell maturation, polarization, and transport functions [38,39]. This improvement could potentially lead to a better removal of uremic retention solutes and a shorter dialysis time. Future studies will be directed towards studying a bidirectional flow, which requires a novel microfluidic design and its optimization. Second, only one flow rate was applied in the functional studies, which was carefully selected based on our previous study [40], building upon prior work [41]. However, it is important to acknowledge that different flow rates should be explored in future studies, as they could potentially impact the clearance capacity [42,43]. To achieve a more accurate representation of physiological conditions, both basal (inner) and apical (outer) flow rates should be fine-tuned and coordinated effectively. By ensuring optimal flow conditions, we can better mimic physiological conditions and ensure an optimal cell behavior and respective transport function. This is of particular importance since toxin clearance can be affected by the flow rate of the blood/plasma and DF near the membrane surface, which will require further optimization to avoid toxin accumulation, thus reducing its clearance [44]. Subsequently, enhancing PBUT clearance could lead to a reduction of the BAK size, enabling the development of portable or even wearable versions of the device. Third, perfusion times of only 30 min were evaluated and experiments with longer durations should be tested to have a thorough understanding of the tubule’s capability. Furthermore, extending perfusion time will also require an optimization of the device itself, as continuous perfusions are prone to air bubble entrapment, leakage, and potential cell detachment [45,46]. Nonetheless, prolonged exposure to DF might compromise cell homeostasis, although we found a 4 h exposure to be within the accepted limits.

In conclusion, the findings from this study demonstrate that the use of DF does not have a detrimental impact on PTECs’ viability or their ability to secrete PBUTs. Furthermore, the maintained barrier function and clearance capacity of the bioengineered kidney tubules as a functional unit of BAK provides a solid base and paves the way for its further development. This includes an upscaling of the current model, by including multiple fibers and other features mentioned above, and its preclinical validation. Altogether, our model serves as a valuable tool for investigating novel dialysis strategies.

## 4. Materials and Methods

### 4.1. Reagents

The DF was freshly prepared by adding sodium hydrogen carbonate (Fresenius, Zeist, The Netherlands) to an acid concentrate for a bicarbonate dialysis (MTN, Neubrandenburg, Germany) followed by a dilution in demi water. The final composition of th eDF is provided in Supplementary Table S1. The DF was used within 24 h of preparation. The concentration of a panel of 7 PBUTs (indoxyl sulfate, indoxyl-β-d-glucuronide, indole-3-acetic acid, kynurenic acid, l-kynurenine, hippuric acid, p-cresylsulfate, p-cresylglucuronide), healthy and uremic plasma, HP, and UP used within this study is available in Supplementary Table S2. Chemicals were purchased from Sigma-Aldrich (Zwijndrecht, The Netherlands) unless stated otherwise. Of note, HSA (A9511, Sigma Aldrich) used in this study was not further purified, hence minor impurities (e.g, fatty acids) could still be in its composition. Additionally, we confirmed that 1 mM HSA/Krebs-Henseleit Buffer supplemented with 10 mM HEPES (KHH buffer, pH 7.4) and used in Section 4.5.’s tubules did not contain considerable amounts of the PBUTs tested.

Flat type 2 F iv hydrophilic polyethersulfone micromembranes (microPES, thickness 100 μm, max pore size 0.5 μm) and microPES type TF10 hollow-fiber capillary membranes (wall thickness 100 μm, inner diameter 300 μm, max pore size 0.5 μm) were obtained from 3M GmbH (Wuppertal, Germany). HP and UP were obtained from the donor minibank of Utrecht University Medical Center (Utrecht, The Netherlands), with informed consent and donor anonymity ensured by internal protocols.

### 4.2. CiPTECs-OAT1

Conditionally immortalized proximal tubule epithelial cells overexpressing the organic anion transporter 1 (ciPTECs-OAT1) were cultured as reported previously [7]. Briefly, cells were cultured up to 60 passages in Dulbecco’s Modified Eagle Medium/Nutrient Mixture F-12 (1:1 DMEM/F-12) (Gibco, Life Technologies, Paisley, UK) supplemented with 10% fetal calf serum (FCS) (Greiner Bio-One, Alphen aan den Rijn, The Netherlands), 5 μg/mL insulin, 5 μg/mL transferrin, 5 μg/mL selenium, 35 ng/mL hydrocortisone, 10 ng/mL epidermal growth factor, and 40 pg/mL tri-iodothyronine, or a complete culture medium. Cells were cultured at 33 °C and 5% (*v*/*v*) CO_2_ to allow expansion.

### 4.3. Evaluation of the Effects of DF on Flat Cultures of ciPTECs-OAT1

#### 4.3.1. Two-Dimensional Exposure of Cells to DF

ciPTEC-OAT1 cells were seeded at a density of 63,000 cells/cm^2^ in 96-well plates. Subsequently, cells were grown for 24 h at 33 °C, 5% (*v*/*v*) CO2 to allow adhesion and proliferation, then transferred to 37 °C, 5% (*v*/*v*) CO_2_ where they were cultured for 7 days to allow for differentiation and maturation, refreshing the medium every other day. The temperature shift ensured the maturation of cells into fully differentiated epithelial cells able to form confluent monolayers. After this period, the cells were carefully washed with Hank’s balanced salt solution (HBSS, Life Technologies Europe BV, Roskilde, Denmark) and subsequently incubated into 100 μL of DF/well for up to 240 min (the standard dialysis time). At selected time points, several assays that reflected the viability and functional performance of the cells were performed, as described below.

#### 4.3.2. Cell Viability Assay

Cell viability was measured using the PrestoBlue^®^ cell viability reagent (Life Technologies). After exposure to DF, the cells were rinsed once with HBSS and incubated with 100 μL/well of PrestoBlue^®^ cell viability reagent (diluted 1:10 in complete culture medium), in the dark. After 1 h of incubation at 37 °C, 5% (*v*/*v*) CO_2_, 80 μL was transferred from each well into a separate 96-well plate. The fluorescence was measured using a GloMax^®^ Discover microplate reader (Promega, Madison, WI, USA), at an excitation wavelength of 530 nm and emission wavelength of 590 nm. Data were corrected for the background, normalized to untreated cells, and presented as relative percentage (%) of viability of untreated cells.

#### 4.3.3. Intracellular Reactive Oxygen Species (ROS) Detection

Intracellular ROS generation was measured by means of cell-permeant fluorogenic substrate 2′,7′-dichlorofluorescein diacetate (H2DCFDA). Briefly, cells were washed once with HBSS, immediately loaded with H2DCFDA (50 μM in serum-free medium) and incubated at 37 °C, 5% (*v*/*v*) CO_2_, in the dark for 45 min. Afterwards, cells were washed with complete culture medium and exposed to DF for several periods of time, up to 240 min at 37 °C, 5% (*v*/*v*) CO_2_, in the dark. H_2_O_2_ (500 µM) was used as a positive control. Following the incubation, cells were washed twice with HBSS and lysed using 0.1 M NaOH for 10 min. Finally, fluorescence was measured at an excitation wavelength of 490 nm and emission wavelength of 520 nm, in a GloMax^®^ Navigator microplate reader (Promega, Madison, WI, USA). Measured fluorescence values were corrected for the fluorescence of the blank sample (untreated lysed cells) and used to calculate relative ROS production, using untreated cells as control.

#### 4.3.4. Lactate Dehydrogenase (LDH) Activity

The evaluation of cell membrane integrity upon exposure to DF was evaluated by measuring the extracellular LDH activity. LDH is an intracellular enzyme which catalyzes NADH lactate to pyruvate. LDH is released into the supernatant from the cytosol upon cell damage. Briefly, upon exposure to DF, samples were collected on ice. The LDH activity was measured using the Cytotoxicity Detection KitPLUS (Roche, Mannheim, Germany), following the manufacturer’s protocol. Briefly, 50 μL of cell supernantant was added to a 96-well plate. In addition, a calibration curve using an NADH (1.25 mM) standard was prepared. The assay buffer was added to a final volume of 50 μL per well, and then a master reaction mix was added in each well (1:1, *v*/*v*). After 10 min, the absorbance was measured at 450 nm in a GloMax^®^ Navigator microplate reader (Promega, Madison, WI, USA), and every 10 min until the absorbance measured in a sample was higher than the highest level of NADH in the calibration curve (12.5 nmol/well). The extracellular LDH activity was expressed as a percentage (%) of the positive control at the corresponding time point. Triton X-100 (Merck, Darmstadt, Germany) at 0.5% (*v*/*v*) was used as positive control and untreated cells as negative control.

#### 4.3.5. IL-6 and IL-8 Release

The release of IL-6 and IL-8 upon exposure to DF was measured using the enzyme-linked immunosorbent assay (ELISA). Cell culture supernatants were collected after 240 min of exposure. Afterwards, cell culture supernatants were centrifuged for 10 min, 240× *g*, 4 °C, and stored at −20 °C until analysis. DuoSet^®^ ELISA Development Systems kits (IL-6 and IL-8, R&D Systems, Abingdon, UK) were used to quantify the cytokines levels in the supernatants following manufacturer’s instructions. The optical density was determined using a GloMax^®^ Navigator microplate reader (Promega, Madison, WI, USA) set to 450 nm. Untreated cells, cultured in serum-free medium, were used as negative controls, while exposure to lipopolysaccharide (LPS, *Escherichia coli* 0127: B8, 10 μg/mL) was used as positive control for the release of IL-6 and IL-8 [24].

#### 4.3.6. OAT1-Mediated Fluorescein Uptake

To evaluate the effect of the DF on OAT1 activity, we performed an OAT1-mediated fluorescein uptake assay. Upon exposure to DF, ciPTECs-OAT1 were carefully washed with HBSS and then incubated with fluorescein (1 μM) prepared in KHH buffer for 10 min at 37 °C. To confirm the activity of OAT1, probenecid (500 μM in KHH) was simultaneously incubated with fluorescein. Uptake arrest was performed by washing the monolayers with ice-cold HBSS, and then the cells were lysed by 100 μL 0.1 M NaOH for 10 min, at room temperature (RT) and under mild shaking. Intracellular fluorescence was detected using a GloMax^®^ Navigator microplate reader (Promega, Madison, WI, USA), at an excitation wavelength of 490 nm and an emission wavelength of 520 nm. Untreated cells were used as positive control. Data are represented as a percentage (%) of positive control.

### 4.4. Combinatorial Effect of DF and Plasma on ciPTECs-OAT1 Seeded onto Transwell^®^ Inserts

#### 4.4.1. CiPTECs-OAT1 Culture on Adapted Transwell^®^ Inserts

To address the basal exposure to plasma and the apical exposure to DF, we cultured ciPTECs-OAT1 cells on an adapted Transwell^®^ (Corning Costar, Corning, NY, USA) system in which the membranes were replaced with a flat microPES membrane with the same characteristics (porosity, pore size and thickness) as those of the HFM used for dialysis. For this purpose, round-shaped pieces of the microPES membranes (diameter, 12 mm; surface growth area, 1.12 cm^2^) were cut from the flat sheets, mounted on empty Transwell^®^ membrane support systems using custom-made sealing rings [47], sterilized with 0.2% (*v*/*v*) solution of peracetic acid (Sigma Aldrich, Zwijndrecht, The Netherlands) in 4% (*v*/*v*) ethanol for 1 h, and then extensively rinsed with HBSS. Afterwards, to ensure cell attachment and growth, a double coating was applied on the membranes based on a previously reported protocol [47].

First, the membranes were incubated with sterile 2 mg/mL L-DOPA (L-3,4-dihydroxyphenylalanine, Sigma Aldrich, Zwijndrecht, the Netherlands) prepared in 10 mM Tris buffer (pH 8.5) at 37 °C for 4 h. The second coating consisted of a 25 μg/mL solution of collagen IV (Sigma Aldrich, Zwijndrecht, the Netherlands) for 1 h at 37 °C. Following the coating procedure, microPES membranes were washed in HBSS and used further for cell seeding. ciPTECs-OAT1 were seeded on double-coated membranes in the apical compartment of the Transwell^®^ system at 90,000 cells/cm^2^.

#### 4.4.2. Exposure to Plasma and Assessment of Monolayer Permeability

After initial proliferation at 33 °C for 3 days and 7 days of maturation at 37 °C, the apical compartment was loaded with 500 μL of DF, while the basal compartment was loaded with 1 mL of HP or UP and incubated for 240 min. Cultures with complete culture medium on both apical and basal sides were used as controls. Following the incubation period, the monolayer permeability was assessed by quantifying the diffusion of the fluorescein isothiocyanate-inulin (FITC-inulin, Sigma Aldrich, Zwijndrecht, The Netherlands) (0.1 mg/mL in Krebs-Henseleit (KH) buffer (Sigma Aldrich, Zwijndrecht, The Netherlands) supplemented with 10 mM HEPES (Acros Organics, Bridgewater, NJ, USA) from the basolateral to apical compartment for 30 min at 37 °C, under gentle shaking. Coated and noncoated membranes without cells were used as controls. Fluorescence was measured at an excitation wavelength of 490 nm and an emission wavelength of 520 nm, by means of a fluorescent plate microplate reader (Promega, Madison, WI, USA). Measured fluorescence values were used to calculate the inulin-FITC concentration in the apical compartment of all samples.

#### 4.4.3. Immunocytochemistry

After the evaluation of the membrane permeability, the cells were washed with HBSS twice, fixed with 4% (*w*/*v*) paraformaldehyde in PBS for 20 min, then washed three times with a 0.1% (*v*/*v*) Tween (Sigma Aldrich, Zwijndrecht, the Netherlands) solution in PBS and permeabilized with a 0.3% (*v*/*v*) Triton ×100 (Merck, Darmstadt, Germany) solution for 10 min. After another three washing steps with 0.1% (*v*/*v*) Tween-PBS, cells were exposed to a blocking solution (2% (*v/v*) FCS, 2% (*w/v*) bovine serum albumin (BSA), 0.1% (*v/v*) Tween-20 in HBSS) for 30 min, followed by the incubation with Phalloidin-iFluor 594 (1:1000 in blocking solution) (Abcam, Amsterdam, The Netherlands) for 1 h at RT to stain for actin filaments. Finally, membranes were mounted on glass slides using the ProLong Gold antifade reagent containing DAPI (Life Technologies, Eugene, OR, USA), and cells were imaged using a confocal microscope (Leica TCS SP8 X, Leica Microsystems CMS GmbH, Wetzlar, Germany) and analyzed using Leica Application Suite X software 1.4.4 (Leica Microsystems CMS GmbH). The cell coverage was assessed using ImageJ’s analysis and reported as a percentage (%) of coverage of phalloidin staining from the total imaged area.

### 4.5. Assessment of Transepithelial Transport of PBUTs on Bioengineered Kidney Tubules

#### 4.5.1. Culture of ciPTECs-OAT1-Seeded HFM, Perfusion with Plasma, and PBUTs Quantification

To assess the transepithelial transport of PBUTs from the basal compartment into the apical side containing DF, we employed a perfusion setup consisting of HFM coated, seeded, and cultured with ciPTEC-OAT1, previously described by Jansen et al. [7]. In this system, HFMs measuring 1.5 cm in length, seeded with mature ciPTEC-OAT1, were loaded into a custom-made perfusion system. The apical compartment was filled with 300 μL of DF. Using a perfusion pump, the HFMs were continuously perfused (6 mL/h) at the basolateral side with KHH (with and without supplementation with 1 mM human serum albumin, HSA) spiked with seven representative PBUTs at concentrations found in CKD patients (Supplementary Table S2) for 30 min at RT, after which the total volume of the apical compartment was collected. In a similar fashion, the fibers were perfused with either HP or UP, respectively. Before perfusion, both HP and UP were passed through a 40 μm strainer to remove protein clumps that could obstruct the fibers. The albumin concentration in UP was found to be 0.70 mM.

Total PBUTs’ concentrations (free + protein-bound) in the DF compartment were analyzed using a LC-MS/MS method [24,48]. Their transepithelial clearance was calculated according to Equation (1):

Transepithelial clearance = (U × V)/(P × T × A)(1)

where U = apical concentration (μmol/L); V = volume in the apical compartment (mL); P = basolateral concentration (μmol/L); T = time (min); and A = outer surface area (cm^2^).

Using the same LC-MS/MS technique, we also confirmed that the 1 mM HSA/KHH did not contain considerable amounts of the PBUTs studied.

#### 4.5.2. Assessment of Monolayer Integrity following Perfusion with Plasma

To assess the (maintenance of the) monolayer integrity after the perfusion with plasma, we conducted an evaluation of the FITC-inulin leakage in the apical compartment. The exposed fibers were perfused once again, this time with FITC-inulin (0.1 mg/mL) in KHH for 10 min. Subsequently, we measured the FITC-inulin leakage in the apical compartment, as described above. The data were reported as percentage of leakage compared with the positive control. Fibers without cells were used as positive control for leakage and represented as 100% leakage.

#### 4.5.3. Evaluation of Albumin Leakage

To investigate the potential protein leakage during perfusion with plasma while exposed with DF at the apical side, a similar setup was prepared to evaluate the leakage of HSA from the basal side into the apical side. We collected the entire apical volume at specific time points and replenished it with fresh DF. Protein content was determined using the Bradford assay (BCA Protein Assay Kit, ThermoFischer, Waltham, MA, USA) following the manufacturer’s instructions. Based on the perfusion rate, we calculated the total amount (mg) of HSA perfused through the fibers for the experimental period.

### 4.6. Statistical Analysis

The data and graphs were analyzed and plotted using GraphPad Prism version 9.5.2 (GraphPad Software, Inc., San Diego, CA, USA). Quantitative data are reported as mean ± standard deviation (SD). The statistical test and replicates are indicated in the figure legends. A *p*-value < 0.05 was considered statistically significant.

## Figures and Tables

**Table 1:** Clearance rate of the individual PBUTs obtained after perfusion with either PBUTs, PBUTs containing 1 mM HSA, or UP ^1^.

PBUTs	Clearance Rate (μL/min/cm^2^) Mean ± SD		
	PBUT	PBUT + HSA	UP
Kynurenic acid	29 ± 11	64 ± 11 (****)	27 ± 11
Kynurenine	38 ± 8	73 ± 11 (****)	12 ± 5
Hippuric acid	27 ± 6	37 ± 6 (**)	10 ± 2
Indoxyl sulfate	35 ± 8	79 ± 11 (****)	12 ± 3
Indole-3 acetic acid	27 ± 7	49 ± 7 (****)	47 ± 6
p-cresyl sulfate	27 ± 7	51 ± 7 (****)	11 ± 3
p-cresyl glucuronide	46 ± 20	64 ± 20	30 ± 4

^1^ Clearance values are presented as mean ± SD. ** *p <* 0.01 and **** *p <* 0.0001 (Multiple Mann–Whitney tests, using PBUTs values as controls).

## Data Availability

The data underlying this article will be shared upon reasonable request to the corresponding author.

## Supplementary Materials

The following supporting information can be downloaded at: https://www.mdpi.com/article/10.3390/ijms241512435/s1.
