# Supplementary material for: Bioengineered Kidney Tubules Efficiently Clear Uremic Toxins in Experimental Dialysis Conditions
Source: Int J Mol Sci. 2023 Aug 4;24(15):12435. doi: 10.3390/ijms241512435 (PMC10419568; doi:10.3390/ijms241512435)
Supplement: Supplementary file 1 [file ijms-24-12435-s001.zip › ijms-2521434-supplementary.pdf]

Supplementary Tables

Supplementary Table S1. Dialysis fluid and culture medium solute concentrations

Solute	DF concentration (mmol/L)	Culture medium Concentration (mmol/L)
Na^+	130	121.6
K^+	3.00	4.16
Mg^{2+}	0.50	0.71
Ca^{2+}	1.50	1.05
Cl^-	110	126.1
CH_3COO^-	3.00	0
HCO_3^-	32.0	29.0
Glucose	5.55	17.5

Supplementary Table S2. PBUTs concentrations in healthy individuals, kidney disease patients and as applied within the PBUTs cocktail[1] and in uremic plasma (UP) in this study, with their individual protein binding[2].

PBUTs	Healthy conc. (µM) (Mean ± SD)	Uremic conc. (µM) (Mean ± SD)	PBUTs (µM)[1]	UP (µM)[2]	Protein binding (%)[3]	OAT1 affinity (uM)[4]
Indoxyl sulfate	2.3 ± 18.8	173.5 ± 121.9	100	68.0 ± 5.8	87-98	20.5
p-cresyl sulfate	10.1 ± 12.2	122.2 ± 90.3	500	42.2 ± 2.5	95	232
p-cresyl glucuronide	0.3 ± 0.2	30.1 ± 6.7	40	1.35 ± 0.10	12-13	-
Indol-3-acetic acid	2.9 ± 1.7	11.4 ± 2.3	3	5.28 ± 0.56	53-69	14.0
Hippuric acid	16.7 ± 11.2	608.4 ± 362.8	300	177.2 ± 11.4	39-41	23.5

Kynurenic acid	0.03 ± 0.01	0.8 ± 0.4	3	2.55 ± 0.20	95	5.1
L-kynurenine	1.9	3.3 ± 0.9	5	4.00 ± 0.42	67	-

[1]Concentrations reported on EUTox Uremic Solutes Database (https://database.uremic-toxins.org accessed on 28 March 2023) and Mihajlovic et al. [24]. [2]Albumin concentration in UP (HSA, 0.70 mM). [3]Protein binding values were reported in [28]. [4]OAT1 affinity constants were reported in [4].
